# Cement Embolism After Kyphoplasty

**DOI:** 10.7759/cureus.52821

**Published:** 2024-01-23

**Authors:** Cade R McGarvey, Ajay Nair, Yusuf Nawras, Jared Oenick, Venkatramana R Vattipally

**Affiliations:** 1 Neurological Surgery, The University of Toledo College of Medicine and Life Sciences, Toledo, USA; 2 Radiology, Advanced Radiology Services, PC (Professional Corporation), Grand Rapids, USA

**Keywords:** revision balloon kyphoplasty, pce, pulmonary cement embolism, balloon kyphoplasty, kyphoplasty

## Abstract

Kyphoplasty is used for the treatment of vertebral compression fractures. The procedure involves inflating a balloon at the compression site; then, polymethylmethacrylate (PMMA) cement is added into the space created by the balloon, where it polymerizes, achieving stabilization, with possible expansion of the vertebral angle. The process is guided by X-rays. Complications are rare, especially when compared to vertebroplasty, and one rare complication is pulmonary cement embolism (PCE). Although many cases are likely undetected due to a lack of symptoms, symptomatic cases require treatment, as they can sometimes prove fatal. We present a case of a patient who underwent kyphoplasty and later presented with a PCE. The PCE was diagnosed using X-rays and computed tomography (CT).

## Introduction

Kyphoplasty is a procedure used to treat vertebral compression fractures. Vertebral compression fractures are common in cases of trauma, cancer, and osteoporosis [1]. Osteoporosis compression fractures typically occur near the thoracolumbar junction. They often follow ankle, wrist, or hip fractures. Kyphoplasty stabilizes, and may even re-expand, the vertebral body through the injection of polymethylmethacrylate (PMMA) into the collapsed vertebral body [2]. Under X-ray guidance, a balloon is placed into the compression site, inflated, and the PMMA cement rapidly polymerizes following injection [1]. After the procedure, the vertebral structure is stabilized, and even improved, though not completely restored. Though further damage from the compression fracture can be prevented, the vertebrae will not be restored to their pre-compression fracture strength and size [3].

Pulmonary cement embolism (PCE) is a potential complication of kyphoplasty, where the cement leaks out of the vertebrae and travels through the venous plexus or arterial vessels [1]. This typically occurs when the PMMA possesses too low a viscosity, when it is subjected to excessively high pressure, when the vertebrae are overfilled, due to variations in needle position relative to basivertebral veins, or due to insufficient PMMA polymerization time [1,4]. Complications can include cardiac tamponade, cardiac perforation, and cement implantation syndrome [1]. Few cases of PCE have been described, and most of them were non-fatal [4]. We report a case of a patient who presented with a PCE following kyphoplasty.

## Case presentation

**Figure 1:**
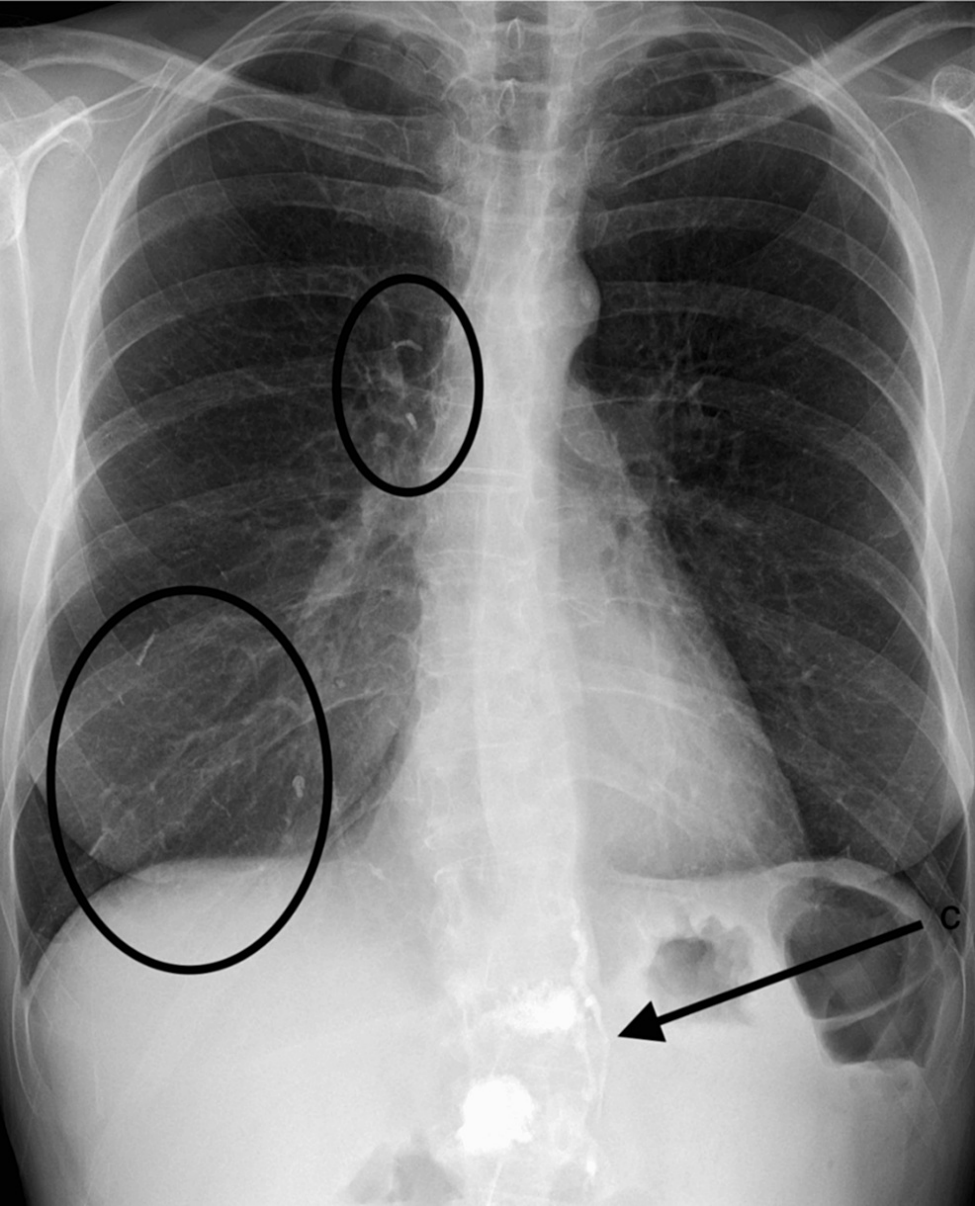
Chest X-ray. The arrow shows polymethylmethacrylate (PMMA) extravasation in the paravertebral veins. The circles show embolization of the lungs.

**Figure 2:**
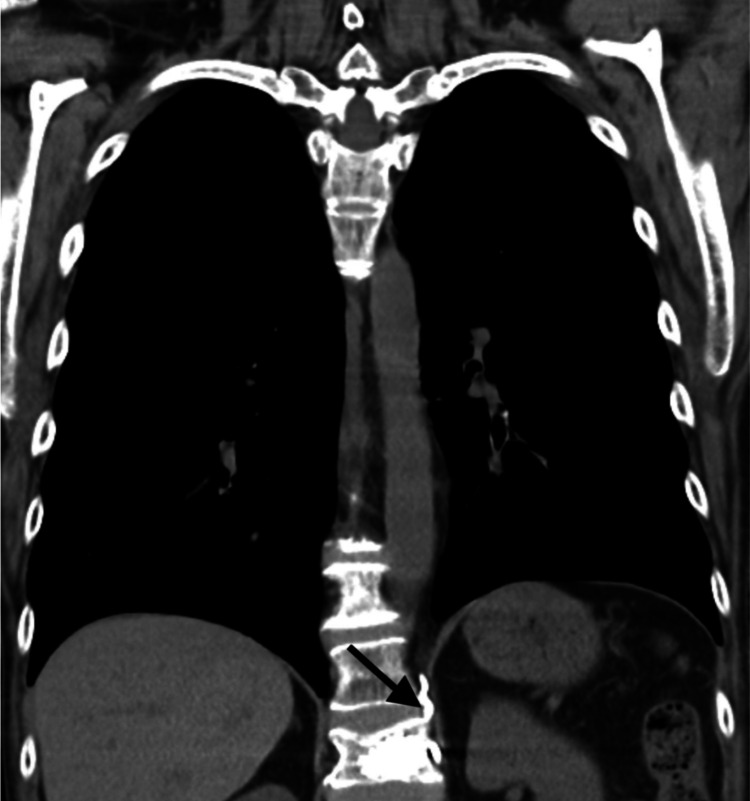
CT showing extravasation of polymethylmethacrylate (PMMA) in paravertebral veins as indicated by the arrow.

**Figure 3:**
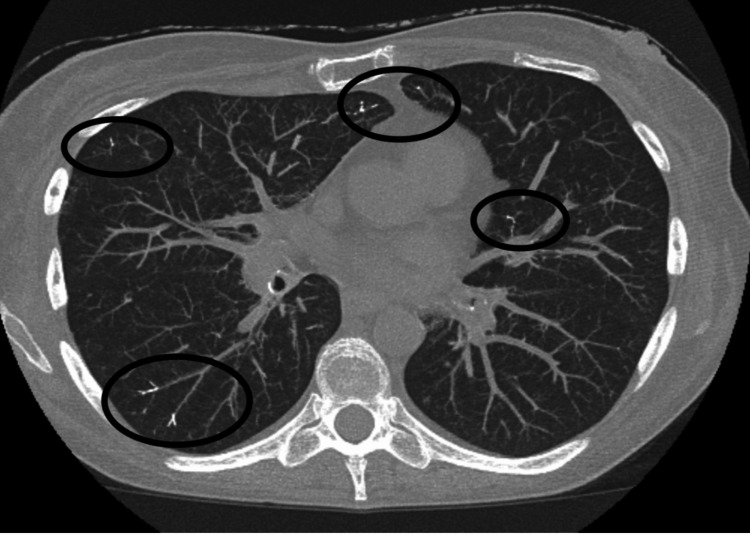
CT showing embolization of the lungs as indicated by the circles.

A 55-year-old woman presented to the ED with complaints of shortness of breath. Her prior medical history was significant for smoking, COPD, and osteoporosis. Several weeks prior to presentation, she developed osteoporotic compression fractures of the first and second lumbar vertebrae. She underwent subsequent kyphoplasty for vertebral stabilization and pain control. The shortness of breath started soon after the procedure. Laboratory findings were within normal limits, ruling out infectious and cancerous etiologies. Radiographic and computed tomography (CT) imaging demonstrated extravasation of PMMA into paravertebral veins (Figure 1: arrow, Figure 2: arrow) and embolization to the lungs (Figure 1: ovals, Figure 3: ovals).

Kyphoplasty is an intervention used to stabilize the vertebral body and reduce pain after a compression fracture. Asymptomatic embolism does not require treatment; there is no standardized treatment for cement embolism, although anticoagulation and surgical evacuation have been documented. Cement embolization is a known complication of kyphoplasty [1].

## Discussion

In rare cases, kyphoplasty can cause complications. These include cement leakage, PCE, new fractures at adjacent levels, allergic reactions, and paralysis. Cement leakage with subsequent pulmonary embolism is the most common complication [4]. There has also been a noted need for opioid prescription fills, similar to patients treated conservatively [5]. Cement leakage results from incomplete PMMA polymerization, which is typically asymptomatic, although it can cause pain if the leakage reaches a nerve root. A PCE occurs when the leakage settles in the lungs [1]. The risk of cement leakage is low compared to vertebroplasty because of both the lesser pressure employed and the increased viscosity of the PMMA used in kyphoplasty [2,4]. Paralysis is rare but can occur from nerve root or spinal cord damage, most often resulting from mispositioning [2-5]. Allergic reactions can occur in response to the PMMA or the iodinated contrast agent used for balloon visualization in X-rays (gadolinium agents can be used in these cases) [6]. Compared to vertebroplasty, kyphoplasty has a lower rate of complications. However, there is a greater monetary cost and longer operation time for kyphoplasty [2-5].

PCE is one of the main risk factors of kyphoplasty because of this phenomenon. Diagnostic imaging predicts a PCE prevalence of 2-26%, though the exact number is unknown. The true number is likely higher, as many patients are asymptomatic, and many symptomatic patients do not undergo thoracic imaging. Most PCEs occur within days to weeks of the procedure, although it is possible to occur immediately after a procedure. PCE can be detected by radiography or computed tomography (CT) imaging [1]. While most commonly asymptomatic, patients with a PCE can present with respiratory complications such as pain while breathing, coughing, or sneezing, and, in rare cases, death [1,2,4]. Although there is no standard treatment for PCE, low-molecular-weight heparin with warfarin or embolectomy can help [1,7].

## Conclusions

Although rare, PCEs are potential complications of kyphoplasty that need to be monitored. Early detection is critical, especially with no standard treatment. PCEs are rarer in kyphoplasty than in vertebroplasty, but they are still possible. We presented a case of PCE following kyphoplasty, diagnosed with radiographic imaging and CT. Patients need to be monitored for the detection of post-operative PMMA leakage, which can cause potentially lethal symptoms. Current treatments are similar to those for pulmonary embolism caused by blood clots. While asymptomatic PCEs do not require treatment, symptomatic PCEs can be potentially fatal if left untreated.
